# Supplementary material for: Multilevel selection analysis of a microbial social trait
Source: Mol Syst Biol. 2013 Aug 20;9:684. doi: 10.1038/msb.2013.42 (PMC3779802; doi:10.1038/msb.2013.42)
Supplement: Supplementary Information — Supplementary figures S1-5 [file msb201342-s1.pdf]

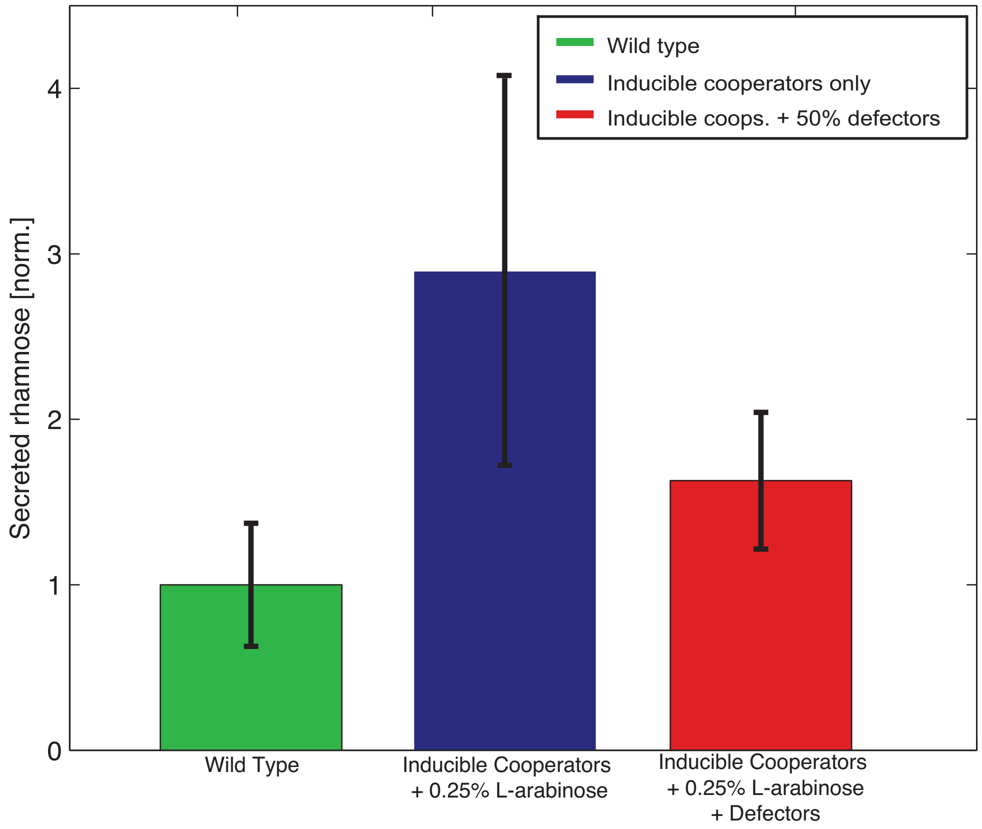

Supplementary Figure S1. Rhamnolipid quantification from inducible cooperators in the presence and absence of defectors shows that surfactant production is unchanged by non-producers. Rhamnose quantification of inducible cooperators alone and in a mix with 50% defectors shows that a population of the inducible strain produces a significantly different amount of rhamnolipids when compared to a mix with defectors (Wilcoxon rank-sum, p=0.028). However, comparing half of the rhamnose production by the inducible alone to rhamnose production in the inducible mix with 50% defectors revealed no significant difference (p=0.382). Rhamnose levels are normalized by production by wild-type cells. Experiments where carried out in liquid culture

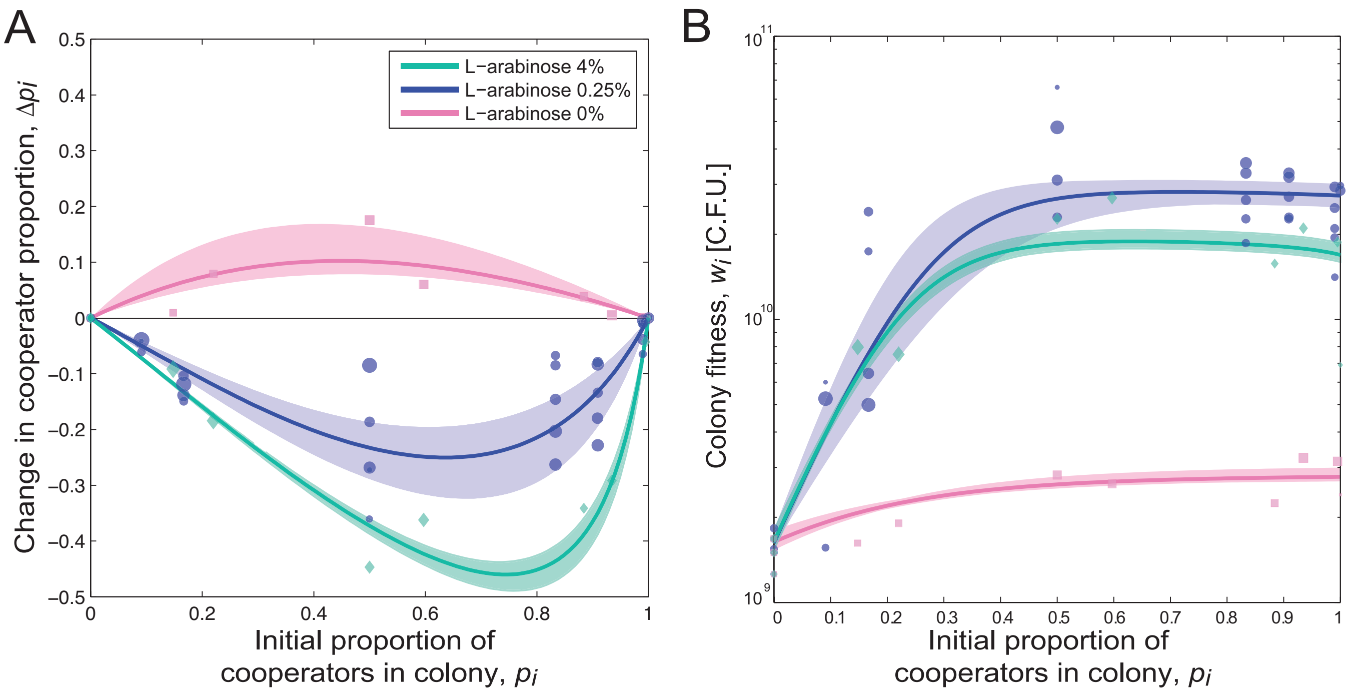

Supplementary Figure S2. Individual-level selection in swarming competitions carried out at three levels of biosurfactant induction. (A) The change in cooperator proportion shows that induced biosurfactant secretion (L-arabinose 0.25% and 4%) strongly selects against cooperation while small production resulting from leaky expression favors cooperators marginally (0%). (B) The colony productivity in the same swarming competitions increases with the proportion of cooperators and depends on the level of surfactant induction.

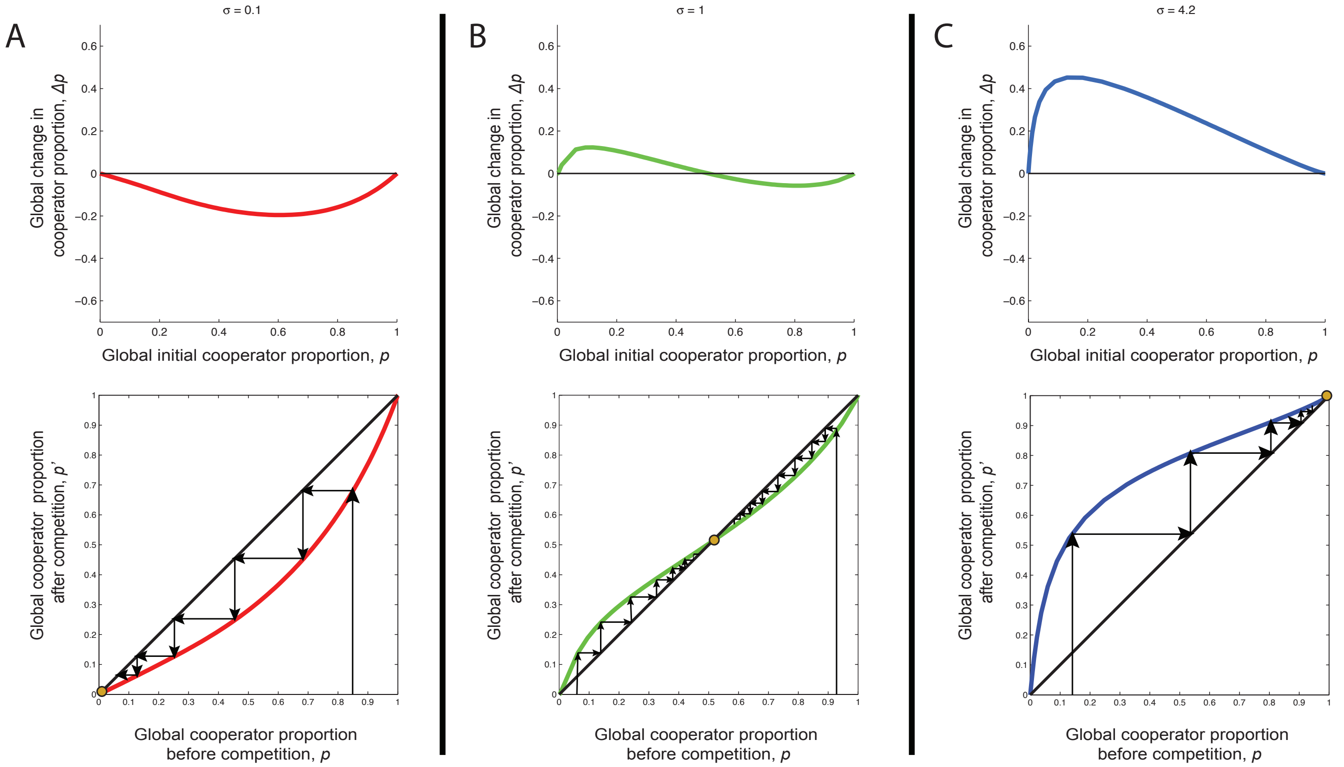

Supplementary Figure S3. Competition dynamics in multilevel selection. (A) Costly swarming cooperation is disfavored when the variance in the distribution of subpopulations is low such as when σ=0.1. The top panel shows the change in cooperator proportion and the bottom panel shows the evolutionary dynamics. In this case the repeated rounds of competition would lead towards extinction of cooperators. (B) Intermediate levels of relatedness (e.g. σ=1.0) shown here) show that cooperation is favored when the cooperator proportion is low, but that cooperation is disfavored when cooperator proportion is high. This phenomenon is due to diminishing returns (fig. 3B) and produces and evolutionary equilibrium where cooperators and defectors coexist (bottom panel). (C) In order for cooperation to be favored all the way to fixation the variance in seeding subpopulations must be significantly high, such as σ=4.2.

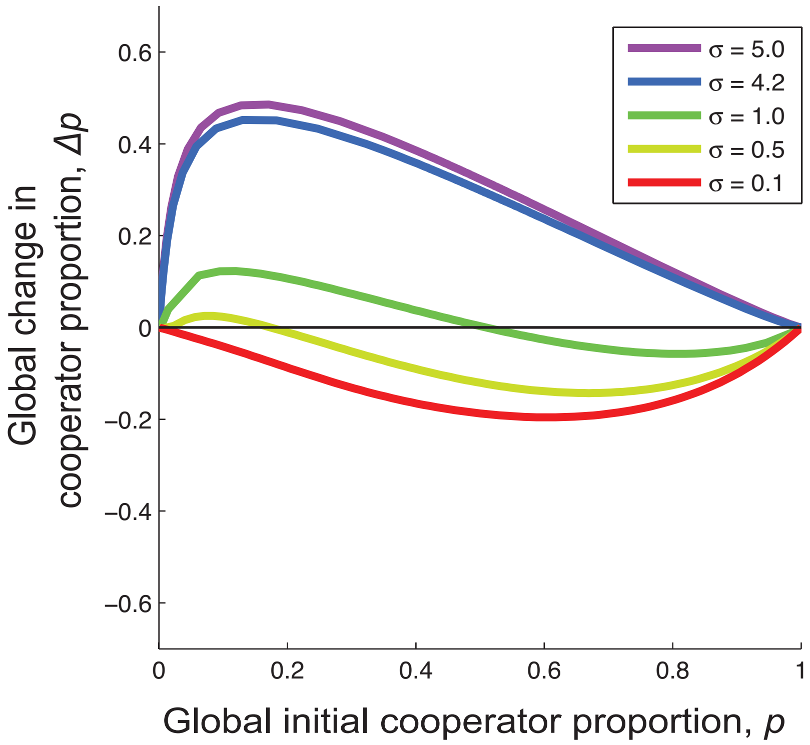

Supplementary Figure S4. Multilevel selection for swarming cooperation is robust to longer competitions. Multilevel selection was analyzed for competitions lasting 48 hours (after population has reached stationary phase) as opposed to 24 hours. Results are unchanged compared with fig. 4A.

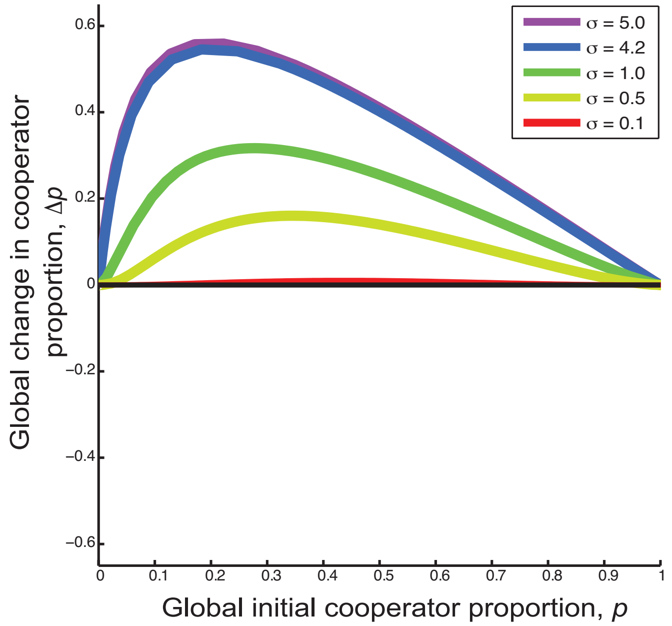

Supplementary Figure S5. Prudent cooperators are favored after multilevel selection independently of individual-level benefit. Multilevel selection was analyzed in the scenario where wild-type cells had no advantage at the individual level and therefore had the *same* yield as defectors, $Y_{WT}=Y_D$. Wild-type strain would still be favored by multi-level selection.
