# Supplementary material for: Multilevel selection analysis of a microbial social trait
Source: Mol Syst Biol. 2013 Aug 20;9:684. doi: 10.1038/msb.2013.42 (PMC3779802; doi:10.1038/msb.2013.42)
Supplement: Supplementary Table 1 [file msb201342-s2.docx]

|  | **q**  **(assumed constant)** | **Y_C_** | **Y_D_**  **(assumed constant)** | **N_max_ (C.F.U.)** | **N_min_ (C.F.U.)**  **(assumed constant)** | **g** | **Pop_0_ (C.F.U.)**  **(assumed constant)** |
| --- | --- | --- | --- | --- | --- | --- | --- |
| **Induc. Strain + 0.25%**  **L-arabinose** | **1** | **0.95**  **CI=[0.93 0.97]** | **1.02**  **CI= [1.01 1.03]** | **2.87**$\boldsymbol{\times}\boldsymbol{10}^{\boldsymbol{10}}$  **CI=**$\boldsymbol{10}^{\boldsymbol{10}}\boldsymbol{\times}$ **[2.6 3.15]** | **1.59**$\boldsymbol{\times}\boldsymbol{10}^{\boldsymbol{9}}$  **CI=**$\boldsymbol{10}^{\boldsymbol{9}}\boldsymbol{\times}$ **[1.49 1.75]** | **10.72**  **CI= [8.1 13.1]** | **3.273**$\boldsymbol{\times}\boldsymbol{10}^{\boldsymbol{4}}$  **CI=**$\boldsymbol{10}^{\boldsymbol{4}}\boldsymbol{\times}$ **[2.5 3.5]** |
| **Induc. Strain + 0%**  **L-arabinose** | **1** | **1.06**  **CI=[1.05 1.08]** | **1.02**  **CI=[1.01 1.03]** | **2.65**$\boldsymbol{\times}\boldsymbol{10}^{\boldsymbol{9}}$  **CI=**$\boldsymbol{10}^{\boldsymbol{9}}\boldsymbol{\times}$ **[2.5 2.82]** | **1.59**$\boldsymbol{\times}\boldsymbol{10}^{\boldsymbol{9}}$  **CI=**$\boldsymbol{10}^{\boldsymbol{9}}\boldsymbol{\times}$ **[1.49 1.75]** | **4.86**  **CI= [4.2 5.7]** | **3.273**$\boldsymbol{\times}\boldsymbol{10}^{\boldsymbol{4}}$  **CI=**$\boldsymbol{10}^{\boldsymbol{4}}\boldsymbol{\times}$ **[2.5 3.5]** |
| **Induc. Strain + 4%**  **L-arabinose** | **1** | **0.88**  **CI=[0.87 0.89]** | **1.02**  **CI=[1.01 1.03]** | **1.95**$\boldsymbol{\times}\boldsymbol{10}^{\boldsymbol{10}}$  **CI=**$\boldsymbol{10}^{\boldsymbol{10}}\boldsymbol{\times}$ **[1.81 2.09]** | **1.59**$\boldsymbol{\times}\boldsymbol{10}^{\boldsymbol{9}}$  **CI=**$\boldsymbol{10}^{\boldsymbol{9}}\boldsymbol{\times}$ **[1.49 1.75]** | **10.68**  **CI=[10.3 11.8]** | **3.273**$\boldsymbol{\times}\boldsymbol{10}^{\boldsymbol{4}}$  **CI=**$\boldsymbol{10}^{\boldsymbol{4}}\boldsymbol{\times}$ **[2.5 3.5]** |
| **WT** | **1** | **1.07**  **CI=[1.067 1.08]** | **1.02**  **CI=[1.01 1.03]** | **4.2**$\boldsymbol{\times}\boldsymbol{10}^{\boldsymbol{10}}$  **CI=**$\boldsymbol{10}^{\boldsymbol{10}}\boldsymbol{\times}$ **[3.89 4.26]** | **1.59**$\boldsymbol{\times}\boldsymbol{10}^{\boldsymbol{9}}$  **CI=**$\boldsymbol{10}^{\boldsymbol{9}}\boldsymbol{\times}$ **[1.49 1.75]** | **4.37**  **CI= [4.3 4.5]** | **3.273**$\boldsymbol{\times}\boldsymbol{10}^{\boldsymbol{4}}$  **CI=**$\boldsymbol{10}^{\boldsymbol{4}}\boldsymbol{\times}$ **[2.5 3.5]** |

**Supplementary Table 1. Parameter optimization.** The parameters are fit to the data using maximum likelihood estimation. The C.F.U. counts of the populations of defectors and cooperators are described by a Poisson distribution with mean λ. As the model allows us to calculate the final number of defectors and cooperators after competition, a given model parameter set defines a λ. We calculate the probabilities of obtaining a certain C.F.U. value from the data ($x_{i}$) given a λ defined by a parameter set ($\theta=[q Y_{C}Y_{D}N_{MAX} N_{MIN}g {Pop}_{0}]$) for the model, $P\left( x_{i}|\theta\right)$. Next we consider the joint probability for cooperators and defectors, $P\left( x_{C}{,x}_{D}|\theta\right)=P\left( x_{C}|\theta\right)\times P\left( x_{D}|\theta\right)$ which is also called the likelihood function $\mathcal{L(}\theta|x_{C},x_{D})$. Our aim is to find the parameter set $\theta$ that will maximize this likelihood function. Without loss of generality, we can maximizes the logarithm of the likelihood function and define our objective function as $\log_{10} \mathcal{L(}\theta|x_{C},x_{D})$=$\log_{10} P\left( x_{C}|\theta\right)+\log_{10} P\left( x_{D}|\theta\right)$. The optimization was done simultaneously with the data from all the competition experiments, thus producing the parameter set that best fits all experiments. Parameters *q*, *Y_D_*, *N_MIN_* and *Pop_0_* where assumed to be the same across all experiments since these are independent of the cooperator strain used. Confidence intervals were given as minimum and maximum values obtained by bootstrapping.
